# Development of a Living Lab for a Mobile-Based Health Program for Korean-Chinese Working Women in South Korea: Mixed Methods Study

**DOI:** 10.2196/15359

**Published:** 2020-01-08

**Authors:** Youlim Kim, Hyeonkyeong Lee, Mi Kyung Lee, Hyeyeon Lee, Hyoeun Jang

**Affiliations:** 1 College of Nursing Yonsei University Seoul Republic of Korea; 2 Mo-im Kim Nursing Research Institute, College of Nursing Yonsei University Seoul Republic of Korea

**Keywords:** mHealth, living lab, intervention mapping, health promotion

## Abstract

**Background:**

Korean-Chinese (KC) women make up the largest group of female migrants in South Korea. To prevent and manage chronic diseases in middle-aged KC women working full time, it is necessary to develop health promotion programs that utilize an online platform because such a platform would allow individuals to participate in health promotion interventions at their convenience.

**Objective:**

This study aimed to develop a living lab for a mobile-based health (LLm Health) program focused on improving the physical activity and cultural adaptation of KC women workers.

**Methods:**

We used a mixed methods design. Living lab principles were factored into the LLm Health program, including the use of multiple methods, user engagement, multistakeholder participants, real-life settings, and cocreation. The program was developed using the 4 steps of the intervention mapping method: needs assessment, setting of objectives, identification of intervention strategies, and intervention design. Needs assessment was conducted through a literature review, focus group interviews with a total of 16 middle-aged KC women, and an online survey related to health promotion of migrant workers given to 38 stakeholders. KC middle-aged women participated in the early stages of program development and provided the idea of developing programs and mobile apps to enhance physical activity and acculturation. The mobile app developed in the program was validated with the help of 12 KC women and 4 experts, including 3 nursing professors and a professor of physical education. They were asked to rate each item based on content, interface design, and technology on a 4-point scale using a 23-item Smartphone App Evaluation Tool for Health Care.

**Results:**

The LLm Health program comprised a 24-week walking program using Fitbit devices, the mobile app, and social cognitive interventions. The mobile app contained 6 components: a step counter, an exercise timer, an online chat function, health information, level of cardiovascular risk, and health status. The cultural aspects and lifestyles of KC women were accommodated in the entire process of program development. The content validity of the mobile app was found to be 0.90 and 0.96 according to the 12 KC women and 4 experts, respectively.

**Conclusions:**

The mobile app was found to be valid and acceptable for KC women. The living lab approach was a useful strategy for developing a culturally adaptive LLm Health program for KC women workers, leading to their active participation in the overall research process, including needs assessment, program composition, and pre-evaluation.

## Introduction

### Background

Migrant workers have health disparities because of language, cultural barriers, and difficulty accessing medical services [1] and are categorized as a vulnerable group in terms of health care access and treatment [2]. In particular, a study of Korean-Chinese (KC) migrant workers in South Korea showed that the percentage of KC workers with poor perceived health was higher than that of native Koreans [3].

KC migrants make up almost 31% of all migrants in South Korea, with a total population of about 680,000. Moreover, 70.4% of KC women are middle-aged [4]. The most common health issues among middle-aged KC women residing in South Korea are musculoskeletal symptoms, depression, cultural adaptation stress, and cardiovascular risk factors (obesity, hypertension, and diabetes) [5,6]. As middle-aged women make up such a large proportion of KC migrants and are experiencing a profound health transition because of menopause, health promotion interventions targeting this group in particular should be developed and delivered in an accessible manner.

Physical activity is highly recommended for health promotion among middle-aged individuals [7]. Previous studies have shown that walking for about 30 min per day for 5 days a week is effective for reducing the risk of cardiovascular disease [8,9]. It may also help prevent musculoskeletal disorders by reducing joint pain and enhancing muscle strength [10].

Therefore, walking exercise may be an effective intervention for addressing the primary health problems of KC women workers. However, although women tend to recognize that a lack of physical activity is a health risk factor, their actual exercise practice remains low [7]. KC women reported that it is difficult to maintain regular exercise because of a lack of time, motivation, and social support or an unfamiliar working environment [11]. To ensure the sustainability of physical activity promotion programs, and thereby maximize their benefits for disease prevention and health promotion, these programs should be both culturally acceptable and easily accessible for KC women. Given the aforementioned barriers, on-site exercise programs may be inappropriate for KC women workers; home-based or mobile-based interventions seem most suitable, as they can be delivered at a convenient time and place. There is evidence for their efficacy: coaching using SMS (including feedback and facilitation) and app-based health promotion programs aimed at facilitating communication between participants and providers have been found to contribute to improved health performance in various community-dwelling populations [12,13].

In South Korea, almost 91% of the population uses smartphones. Smartphone users install more apps than users of other types of mobile device [14]. Moreover, 88% of migrant workers in Seoul have smartphones [15]. In accordance with the high rates of smartphone and app usage, mobile health (mHealth) has been rapidly gaining attention in the field of health promotion. Researchers have defined mHealth as the use of various apps, such as GPS and Bluetooth technology, as well as the basic utilities (voice calling, SMS) of mobile or wireless devices for the purposes of health and health care [16]. Mobile-based interventions are increasingly being adopted to increase migrants’ access to health services. Such interventions have proven useful for expanding information provision and peer and resource support [17-19].

For exercise interventions to be effective for migrants, activities should be tailored to their particular level of experience and demands. One approach to ensuring such tailored interventions is a living lab. In a living lab, participants and stakeholders conduct research to best represent their needs; it is an innovative activity in which users and stakeholders all actively participate in the research process, even while at home, for the betterment of community problem solving or services [20]. In an integrative review study [21], the living lab approach was identified as an appropriate way to identify and address the health needs of vulnerable groups for whom health care services are economically and locally less accessible. The living lab approach is useful for developing culturally specific health promotion programs centered on KC women through the application of its core principles, namely, user participation, the use of multiple methods, stakeholder participation, basis in real-life settings, and cocreation.

### Objectives

The purpose of this study was to apply the living lab approach to KC women (who are, in this context, the users) to enable their direct participation in the development of a culturally sensitive mobile app–based health promotion program (living lab for a mobile-based health [LLm Health]), alongside community stakeholders. We hope that the program will help contribute to the health promotion activities of middle-aged KC women and establish a culture of health promotion in the community.

## Methods

### Overview

This study used a mixed method design to develop a mobile app–based health promotion program using the living lab approach [22]. Living labs emphasize a multimethod approach, user engagement, multistakeholder participants, real-life setting, and cocreation. In this study, the process of program development was, through the intervention mapping approach [23], as follows: needs assessment, setting of objectives, identification of intervention strategies, and intervention design. This study was approved by our institutional review board (IRB-2017-1641-001).

### Needs Assessment

The needs assessment comprised a literature review, focus group interviews, and a stakeholder analysis.

#### Literature Review

We searched the PubMed, EMBASE, and CINHAL databases for studies published in English up to November 2017. The search terms were “mobile applications and health promotion” OR “smartphone applications AND health promotion” OR “app-based intervention AND health promotion.” We ultimately analyzed 12 out of 191 studies, after excluding those that did not meet the criteria. More specifically, we considered only the studies on health interventions for adults in peer-reviewed journals, having excluded studies that used only SMS text messaging or Web-based interventions.

#### Focus Group Interviews

The focus group interviews were conducted between November and December 2017. A total of 3 interviews were conducted with 16 middle-aged KC women; each interview lasted between 60 and 90 min. The participants were from Seoul and Gyeonggi-do, aged between 40 and 65 years, had been working full time for the last 6 months, were able to communicate in Korean, understood the purpose of the study, and agreed to participate. The interviews were conducted in a church education center in the K district of Seoul, which has a large population of KC women. The interviews were carried out by researchers trained in conducting focus group interviews.

The focus group guideline was developed by the researchers according to the guidelines of Krueger and Casey [24]. Before each interview, participants were informed of the necessity of the research, its purpose and methods, that the data would be anonymous, and that they could withdraw from the interview at any time. The questions asked in the interviews focused on the health information necessary for health promotion of KC women workers and the difficulty of adapting to everyday life as a foreign worker. The interviews were recorded and transcribed and then subjected to content analysis. A researcher repeatedly read the transcripts; identified and verified meaningful words, sentences, and paragraphs; and categorized them into themes.

#### Stakeholder Analysis

An online questionnaire was conducted for the stakeholder analysis, from November to December 2017. In total, 38 people were surveyed as community stakeholders, including those in national institutions (public health centers), local community organizations (Korean support center for foreign workers and KC church), KC merchant society (Koreans who employed KC women), and KC women who participated in the focus group interview. We provided the survey participants with the same information on the study that we provided to the focus group participants (eg, necessity, purpose, and anonymity of data) via SMS text message and obtained their voluntary consent to participate. Stakeholders were provided a URL to the online survey system at the bottom of the explanation comment sent by email. To protect the rights of the participants, the purpose and procedures of the research, privacy protection, and withdrawal from research participation were presented on the start screen before they began the online survey. The online survey was set up so that the survey would proceed step by step only if participants agreed to participate in the survey. The participants completed the online questionnaire, which included 10 items, each rated on a 5-point Likert scale, evaluating the health promotion of migrant workers in terms of interest, importance, influence, and position of stakeholders. The data obtained through the online questionnaires were assigned IDs and stored in an encrypted computer, accessible only to researchers trained in research ethics.

The collected data were analyzed using the Beeye of stakeholder analysis tool.

### Settings of Objectives

The LLm Health program was based on the Interaction Model of Client Health Behavior (IMCHB) by Cox [25]. There are 3 main elements of Cox’s IMCHB: client singularity, client professional interaction, and health outcome. Cox considered that the individual characteristics of subjects, including their socio-psychological factors, are necessary to understand their practice of positive health behaviors, and emphasized the importance of interaction between the participant and provider in determining health behaviors. The element of client singularity is determined by background variables, internal synchronization, cognitive appraisal, and emotional responses. The element of client professional interaction is determined by emotional support, health information provision, decision control, and professional and technical competence. Finally, the element of health outcomes included the use of health care services, clinical health status indicators, health problem severity, and service satisfaction. In this study, we designed various social cognitive interventions that considered the cultural characteristics of participants for inclusion in the LLm Health program. We then examined the effects of intervention on health outcomes to compare the enhanced group with the control group.

### Identification of Intervention Strategies

In this study, we decided on intervention strategies using the behavior change technique (BCT) taxonomy of Michie et al [26]. This taxonomy describes 40 BCTs that have proven effective in producing behavior change, through an analysis of previous studies on behavior change interventions. This strategy is based on a variety of social psychological theories, including social cognitive theory. The main contents of BCTs involve providing information related to the results of the health behavior, setting goals, self-monitoring, overcoming obstacles, feedback, and social support.

### Intervention Design

The LLm Health program was designed to improve the physical activity and cultural adaptation of KC middle-aged women workers. The program consisted of 12-week adaptation and 12-week maintenance phases. Enhanced intervention was designed to strengthen social-cognitive factors related to physical activity for the adaptation phase only. The mobile app was a core intervention tool used through the 24-week period by the participants.

#### Development of the Mobile App

**Figure 1:**
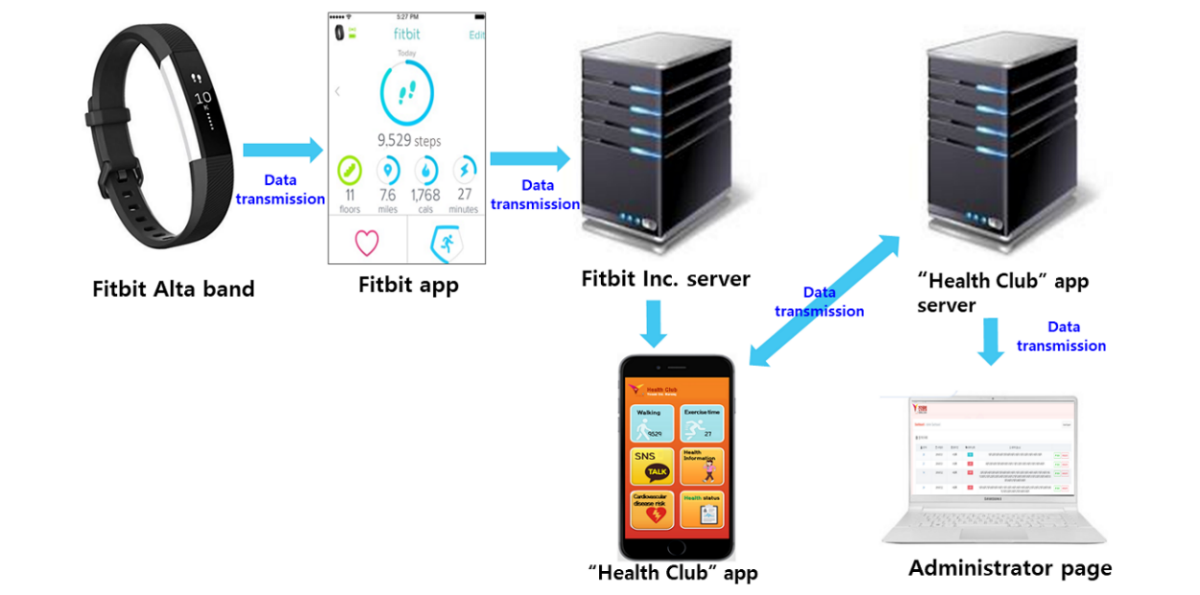
The process of mobile app development.

A total of 10 meetings were held with app developers from November 2017 to February 2018. The mobile app was designed to measure the number of steps and exercise duration of users using smart bands, and we attempted to encourage users to self-monitor their walking adherence through the development of a mobile app linked with the smart bands (Fitbit Alta). The app was designed for the Android operating system based on the resolution of a Samsung Electronics Galaxy S5. Data, such as the number of steps and exercise duration of participants wearing the smart bands, were collected by the app from the Fitbit company server. These data were sent to the mobile app “Health Club” and linked with the administrator Web page for the research team. The data transmission process is shown in Figure 1. To develop a mobile app that suited the participants, we referred to the findings of the needs assessment. Moreover, we consulted with KC participants for help designing the app, color, and logo from the initial development process.

#### Validity Test of the Mobile App

The app was evaluated using the Smartphone App Evaluation Tool for Health Care [27], which contains 23 items in 7 domains: content (accuracy, understanding, and objectivity), interface design (consistency, design suitability, and vocabulary accuracy), and technology (security). Each item was rated on a 4-point Likert scale. The content validity of the app was also evaluated based on the recommendations of at least three experts [28]—specifically, 3 nursing professors with multicultural research experience and a professor of physical education. The content validity was deemed appropriate if the average of the scale-level content validity indexes (the ratio of responders who rated an item with 3 or 4; Scale Content Validity Index/Average) was 0.90 [29].

#### Development of the Enhanced Intervention

The 24-week period was divided into a 12-week adaptation period for providing an intervention and a 12-week maintenance period without the intervention. During the 12-week adaptation period, the following interventions were applied to strengthen the social psychological factors affecting the change in health behavior and social-cognitive capabilities (self-efficacy, social support, etc) of the enhanced treatment group: sending SMS to improve self-efficacy, setting exercise goals, providing feedback, and social networking service (SNS) interaction. In addition, a photovoice activity was planned to represent the exercise promotion factors of community and the sense of local community that KC participants perceive.

The self-efficacy SMS was constructed according to the questionnaire items of the Barrier Self-Efficacy Scale of McAuley et al [30] and the self-efficacy tool of Marcus et al [31].

To help acculturation, offline cultural workshops were planned to provide information about Korean life according to the needs of KC women.

## Results

### Needs Assessment

#### Literature Review

According to the 12 studies on mobile app–based health promotion programs, the primary goals of these programs were physical activity promotion, weight and diet management (8 studies), and establishment of a healthy lifestyle (4 studies). The primary purposes of the apps in these studies were to provide feedback on personal health status (9 studies), behavior change monitoring (9 studies), and health information provision (8 studies). Overall, the mobile app–based interventions for adults without disease were found to be effective for improving health promotion behavior [32].

#### Focus Group Interviews

The analysis of the focus group interviews of middle-aged KC women workers identified the following main topics related to their health management: nutrition management (nutrient proportion and calorie confirmation method), information about chronic diseases, and exercise methods at home. These were, therefore, selected as contents of the LLm Health program. KC women workers also expressed the need for education related to cultural discrimination, such as nonpersonal treatment because of cultural differences in South Korea, the need to learn a newly coined term, and their language restrictions in using English and foreign words. In addition, we confirmed that KC women actively seek health information using smartphones and share this information through online interactions.

#### Stakeholder Analysis

**Figure 2:**
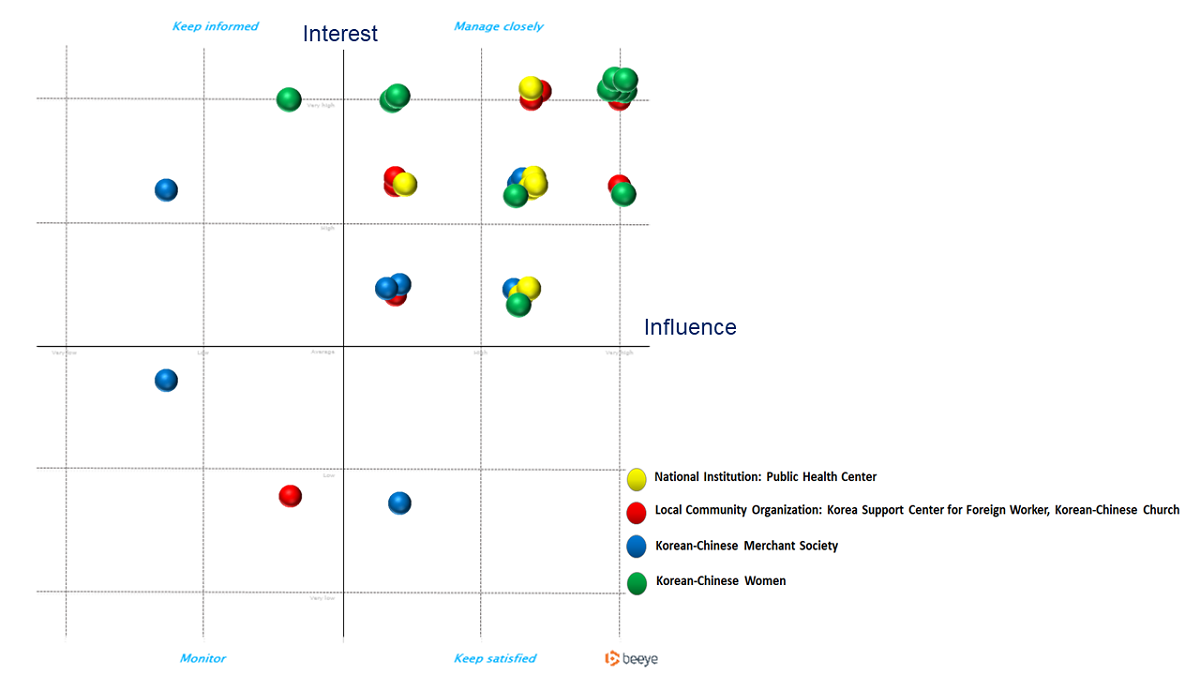
Stakeholder analysis: interest and influence.

Analysis of the stakeholder questionnaire data revealed that the type of participation desired by most stakeholders was a partnership (n=20) in program planning, implementation, and evaluation. Offline health education (n=19), app-based health promotion information (n=11), health information booklets (n=6), and cultural competence training for community practitioners (n=6) were also noted as helpful activities in designing health promotion programs for migrant workers. An analysis revealed that stakeholders from national institutions, local community organizations, and KC women in Korea were highly influential and interested in health promotion of foreign workers, whereas Korean employers showed little interest in it (Figure 2).

### Setting of Objectives

Drawing on the IMCHB of Cox [25], we designed the interaction elements of the participants and experts in the LLm Health program. We decided to deliver health information through health booklets and exercise skills coaching. We used SMS text messages to encourage participants and to motivate them to continue the program as a form of emotional support. Decision control was established by enabling users to identify and implement step-by-step goal setting and monitor the results of follow-up tests in the app. Participants were also encouraged to apply the health information acquired in the offline cultural workshops in their daily life. We ensured access to expertise and technical ability by having the research team be available for counseling regarding the results of participants’ health tests and by providing participants with information on improving and preventing cardiovascular diseases. These interventions were expected to help participants achieve social-cognitive competence (eg, exercise self-efficacy, social support, and community consciousness), leading to an increase in walking adherence.

### Identification of Intervention Strategies

In this study, we applied the BCT taxonomy of Michie et al [26] to the adaptation period (12 weeks after participation in the program) in the enhanced treatment group to strengthen self-efficacy and social support for exercise performance (Table 1). Self-monitoring of walking adherence was encouraged by providing participants with walking exercise results and through goal setting. In addition, a photovoice intervention was employed to overcome obstacles to exercise and to identify promotive factors using community resources (eg, parks and sports facilities). To encourage cultural adaptation in participants, we provided useful information about life in South Korea. In offline cultural classrooms, participants were encouraged to announce their willingness to practice the contents of education and encourage each other to induce positive behavior changes. Finally, we designed a strategy to provide personal counseling on the results of health exams and to provide nursing prescriptions for lifestyle improvement.

**Table 1:** Behavior change theory methods and practical strategies for the living lab for a mobile-based health program.

Determinants	Behavior change technique methods	Strategies
Exercise adherence	Prompt review of behavioral goal behavior modeling; prompt specific goal setting; set graded tasks; prompt self-monitoring of behavior; provision of feedback on performance	Setting step goal (3 times); sending a medal image (3 times); guiding in possible muscular and stretching exercises indoors
Social support	Setting graded tasks; prompt specific goal setting; environmental restructuring; social support; provide general encouragement; prompt self-talk	Setting goal of walking steps (3 times); sending medal image (3 times); photovoice; SNS^a^ interaction
Self-efficacy	Prompt intention formation; environmental restructuring; social support	Exercise self-efficacy SMS (12 times)
Sense of community	Environmental restructuring; social support; provision of general encouragement; prompt self-talk	Photovoice; SNS interaction
Acculturation	Use of imagery; provision of general encouragement; prompt self-talk	Cultural adaptation contents (6 times); offline cultural workshops (3 times)
Health outcome	Provision of general information on behavior	Personalized face-to-face counseling after health examination

^a^SNS: social networking service.

### Design of Intervention

#### Mobile App “Health Club”

The developed mobile app was named “Health Club.” In the cocreation process of the “Health Club” mobile app, the first task for KC women and stakeholders was to give the mobile app a name that could be easily understood and suitable for KC women. Simple and familiar terms were chosen as better than those that were less familiar. Consequently “Health Club” was selected over “Healthy Program” or “Healthy Heart.” In addition, the researchers and KC women jointly reviewed and revised the mobile app menu design and health topics of interest, giving due consideration to KC women’s preference and lifestyles.

Users could search for and download it from the Google Play Store after registering with a Google email address. To encourage participation in the study, we set up automatic transmission of Fitbit Alta data and Health Club app synchronization notifications once a day on the registered participants’ mobile phones.

The app had 6 main functions, all listed on the main screen: number of walking steps, duration of exercise, chatting, health information, cardiovascular disease risk, and health status. The daily number of steps and moderate-intensity exercise time were obtained via synchronization with the Fitbit Alta, and participants were able to input their strength exercise and stretching time during the day. The group chat feature of the app was linked to an SNS (eg, KaKao talk) to encourage information sharing among participants, leading to an increase in emotional and network support. The health information covered 12 topics, including cardiovascular disease, musculoskeletal disease, menopausal symptoms, aging prevention, stress management, pharmacy and hospital available on weekends, weight control, healthy eating, stretching, strength exercise, and cancer screening for women. This information could be accessed as a PDF file or by linking users to related websites when participants pressed the relevant information icon.

**Figure 3:**
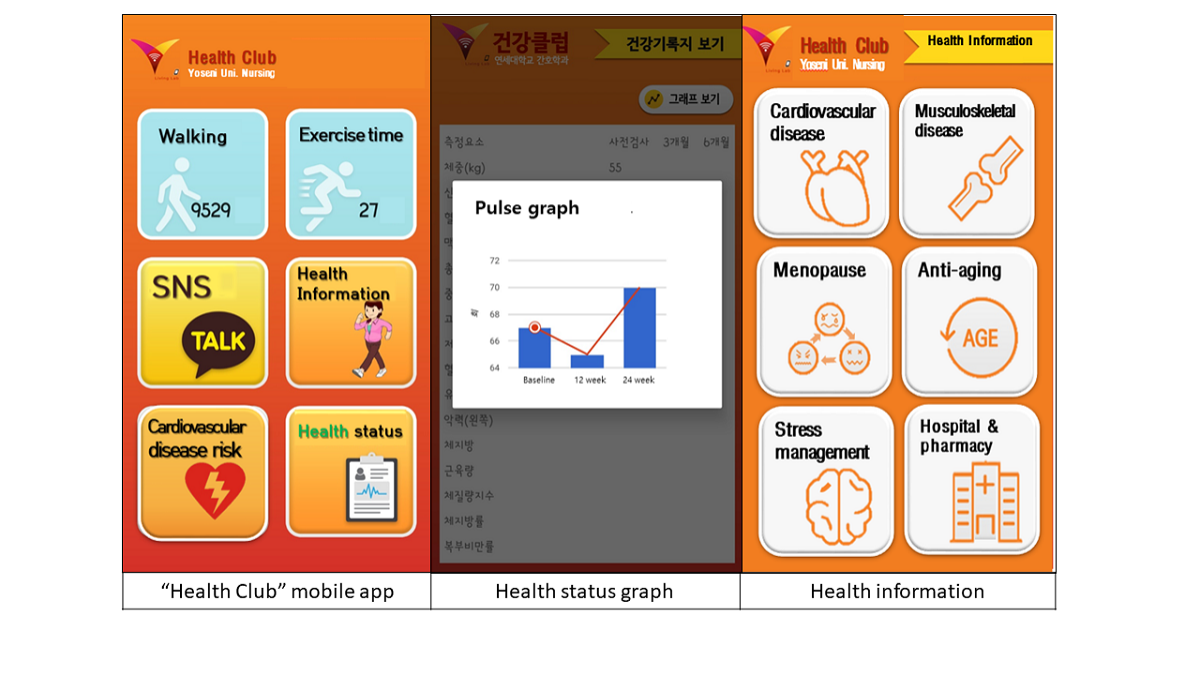
Components of the Health Club mobile app.

Users’ 10-year cardiovascular risk was calculated using an algorithm presented in the Framingham Heart study [33]; it was assessed at baseline and at 12- and 24-week blood tests. The health status menu was designed to check changes in health test results 3 times (baseline, 12 weeks, and 24 weeks) using numerical data and graphs (Figure 3).

#### Pilot Test of the Mobile App “Health Club”

After developing the “Health Club,” we recruited 12 KC middle-aged women for a pilot test of its usability. They were selected as health leaders and were provided with educational materials, the mobile app, and a smart band to play a promotional role in recruiting participants in the following research. After health leaders used the Health Club app for 2 weeks, they were given the app evaluation tool. The content validity of the app was found to be 0.90 according to the 12 KC women. The validity based on responses of the 4 experts to the mobile app was 0.96.

The validity of 7 subitems evaluated by 4 experts and 12 KC middle-aged women were as follows: accuracy=0.90, understanding=0.91, objectivity=0.92, consistency=1, design suitability=0.96, vocabulary accuracy=0.98, and security=0.86. The mobile app was revised and supplemented to reflect opinions on “adding the source of the information” and “adding statements regarding user privacy protection.”

#### Enhanced Intervention

To encourage exercise adherence during the 12-week adaptation period, step goals were set 3 times every 4 weeks, and encouragement with a medal image was sent via SMS whenever participants reached the goal. The app was designed to send a self-efficacy SMS once a week, so that participants could overcome the barriers to exercise and recognize the benefits of exercise. A total of 12 self-efficacy SMS messages were designed, all with less than 60 characters that focused on mood, lack of time, lack of interest, physical discomfort, lack of social support for exercise, limitations because of appearance, limitations of place, fatigue, lack of exercise skills, negative perception of migrant worker exercise, and lack of awareness regarding the benefits of exercise.

The offline cultural classes covered 3 topics: “personal color and makeup,” “low sodium healthy diet,” and “practical English.” These classes emphasized what users could do in real life, and individuals shared their action plans at the end of each class. To confirm the motivational factors and sense of community, participants were encouraged to post 2 pictures taken on the themes of “good exercise in our neighborhood” and “exercise experience with local people” using photovoice.

## Discussion

### Principal Findings

This study developed a culturally appropriate mobile app–based health promotion program for KC women by applying the living lab principles, entitled “LLm Health program.” We also incorporated BCTs [34], which are based on the social cognitive theory of changing health behaviors, as main components of the health promotion program. The app was developed based on the characteristics of KC women—who often experience social isolation [35] and social and cultural challenges (eg, language barriers, changes in socioeconomic status, and emotional difficulties with loneliness and belonging) because of their international migration—to enable them to easily access health information and social support. The Fitbit Alta is a device that can be worn on the wrist by KC women who work in the household and service industries; it allows participants and researchers to monitor real-time exercise adherence.

### Implications

This study is meaningful in that it is the first to apply the living lab approach in the migrant population. The participants were involved in the early stages of program development, proposing ideas for promoting physical activity and cultural adaptation. We believe that participants’ acceptance of the program was enhanced through their participation in deciding the app name, interface design, contents, etc. Moreover, some KC women were selected as community health leaders, who voluntarily participated in the overall research process including program composition, pre-evaluation, and leading living lab–based active participation and cocreation. Through this study, we have involved various community stakeholders, including national institutions and religious facilities, in planning, development, and the implementation of this migrant health promotion program. According to previous studies, the participation of various stakeholders, such as health and welfare agencies, hospitals, clinics, companies, patient associations, and local governments, has been found to be a successful strategy for developing a patient-centered health management model for patients with chronic conditions [36]. In community-based health research, living labs appear to be useful for promoting active health behavior changes, especially among vulnerable social groups who often need to solve their health problems on their own [21]. The LLm Health program was designed to improve middle-aged KC women’s self-efficacy and social support in relation to physical activity, as well as promote their physical, mental, and social health, while considering their specific cultural features. The living lab approach was applied as a platform to help these women solve their health problems by reflecting on their own point of view. In future research, it would be useful to incorporate the living lab principles into existing behavior change theories.

The main component of the LLm Health program was its use of BCTs. Previous studies have found that BCTs can promote the active participation of the vulnerable population. For instance, Sidhu et al [37] applied BCTs to improve the self-management ability of low-income individuals of various races with chronic conditions in the United Kingdom. Mathews et al [38] developed a type 2 diabetes prevention program for low-income individuals in India using BCTs at the individual, interpersonal support, and community levels. A therapeutic intervention using BCTs—primarily goal setting and social support—was an effective strategy for people from low social and economic backgrounds to engage in health promotion behavior [34]. We also applied goal setting [39] (setting a goal for the average number of steps every 4 weeks) in the LLm Health program, as it is well known to help reduce physical activity withdrawal and increase adherence. Developing health programs based on BCT to enhance the capacity of social-cognitive factors, such as the health information provision and SNS interaction used in this study, might be useful in planning effective intervention strategies for groups with different cultural and social backgrounds.

Although this study adopted several BCTs from the list of commonly used BCTs, such as goal setting and behavior modeling used in top-ranked physical activity apps [40,41], it is necessary to thoughtfully consider further BCTs that have been found to be effective for behavior changes in migrant populations.

This study developed a mobile app–based health promotion program that considers the characteristics of middle-aged KC women. Programs to promote migrants’ participation in physical activity should consider the cultural beliefs and constraints of the participants [42]. Middle-aged KC women living in South Korea often live in low-income areas. Moreover, they have poor access to systematic health services [3] or good quality health care and tend to have difficulties with the early detection of illness [5]. They comprise a “culturally and linguistically diverse” population group [43] and experience considerable stress in adapting to Korean society [5]. High acculturation stress is associated with lower physical activity [44]; conversely, efforts to acculturate migrants can positively influence their physical activity levels [45]. It is, therefore, essential to consider the cultural aspects and lifestyles of immigrants when developing intervention programs so that they can best adapt to their new cultures.

From the perspective of convenience—particularly considering that KC women often perform housework and are employed in the service industry—our program relied on data from smart bands (Fitbit Alta), wearable devices that synchronized with the app to allow participants to check their exercise progress directly. Being able to constantly monitor their exercise might have increased their motivation to adhere to the exercise. The use of a smart band, which records objective real-time step counts and moderate-to-vigorous physical activity, overcomes the limitation of a previous study [46] measuring the effects of walking exercise, which relied on self-reported step counts.

This study has several limitations. First, owing to budget constraints, we could not develop an iPhone operating system version of the mobile app; it is only available on the Android operating system. Second, to enable examination of the effect of walking exercise on health, objective data on the number of steps and moderate-to-vigorous intensity exercise time were collected through the Fitbit Alta. However, there is limitation in that strength exercise and stretching had to be self- reported. Third, further efforts are necessary to educate middle-aged KC women on the terminology and technical aspects of wearable and mobile devices, to increase their ease of use. Therefore, it is essential to repeatedly train users in adopting a new technology during the initial adaptation stage.

### Conclusions

We developed a mobile app–based program for the health promotion of middle-aged KC women using the living lab approach, which focuses on expanding participation and strengthening community health capabilities. This approach appears to be useful for creating health promotion program content and intervention strategies. It is recommended that future research examines the effectiveness of mobile app–based health promotion programs on physical and mental health outcomes for KC women.

## Abbreviations

BCT: behavior change technique
IMCHB: Interaction Model of Client Health Behavior
KC: Korean-Chinese
LLm Health: living lab for a mobile-based health
mHealth: mobile health
SNS: social networking service
